# Understanding triglyceride glucose-body mass index: implications for diabetes and cardiovascular disease management

**DOI:** 10.3389/fendo.2025.1675270

**Published:** 2025-09-29

**Authors:** Shunping Zhou, Rong Guo

**Affiliations:** Department of Cardiology, Yangpu Hospital, Tongji University School of Medicine, Shanghai, Shanghai, China

**Keywords:** triglyceride glucose-body mass index, diabetes, cardiovascular disease, insulin resistance, mortality

## Abstract

This article focuses on the Triglyceride-Glucose Body Mass Index (TyG-BMI) in diabetes and its associated cardiovascular complications. It elaborates on the basic concepts, current clinical practices, technological advancements, controversies, challenges, and future directions related to TyG-BMI. By integrating the findings from numerous studies, we aimed to provide a comprehensive understanding of the role of TyG-BMI in diabetes and cardiovascular disease research, diagnosis, and treatment, highlighting its significance and potential for improving the management of diabetic cardiovascular health.

## 1 Introduction

Diabetes mellitus (DM), particularly type 2 diabetes, is one of the most common chronic diseases worldwide (1). The most common complication of diabetes is cardiovascular disease, the leading cause of morbidity and mortality (2). Many biomarkers have been developed to assess the risk of incident diabetic complications; however, few have been validated in subsequent analyses or applied in clinical practice (3). Thus, there is great interest in exploring clinical biomarkers that can accurately predict the risk of diabetes-related diseases and mortality.

Numerous studies have demonstrated the association between the Triglyceride–Glucose (TyG) index and the risk of type 2 DM. For example, in a prospective study of 1923 women and 3016 men from the Vascular Metabolic CUN cohort, the hazard ratio (HR) for incident diabetes per one-standard-deviation increment in the TyG index was 1.54 [95% confidence interval (CI): 1.40– 1.68], indicating that an increase in the TyG index significantly increases the risk of developing diabetes across different metabolic health categories (4). A 4-year retrospective study involving 2900 adults without diabetes found that individuals with a high TyG index had an elevated risk of diabetes compared with those with a low TyG index. For TyG index quartiles, the HRs for quartiles 3 and 4 were 4.06 (p = 0.033) and 5.65 (p = 0.006), respectively, suggesting its predictive value for incident diabetes (5).

Some studies have explored the application of the TyG index combined with anthropometric measures; notably, the Triglyceride–Glucose Body Mass Index (TyG–BMI) has been developed. In a cross-sectional study involving Nigerian adults, TyG–BMI showed good performance in identifying metabolic syndrome (MS). The area under the curve (AUC) for TyG–BMI in detecting MS was 0.838 (95% CI: 0.802–0.870), indicating its potential as a screening tool for MS (6). The relationship between the TyG index and BMI has also been investigated in relation to other diseases. A study on the association between the TyG index and carotid atherosclerosis in patients with non-alcoholic fatty liver disease (NAFLD) found an increased risk for carotid atherosclerosis with an elevated TyG index value. The HR (95% CI) for carotid atherosclerosis risk in the higher TyG index group was significant, with the low TyG index group as the reference. These findings indicate the combined impact of an elevated TyG index and the presence of NAFLD—often related to BMI—on cardiovascular risk (7).

## 2 TyG-BMI: definition

**Figure 1:**
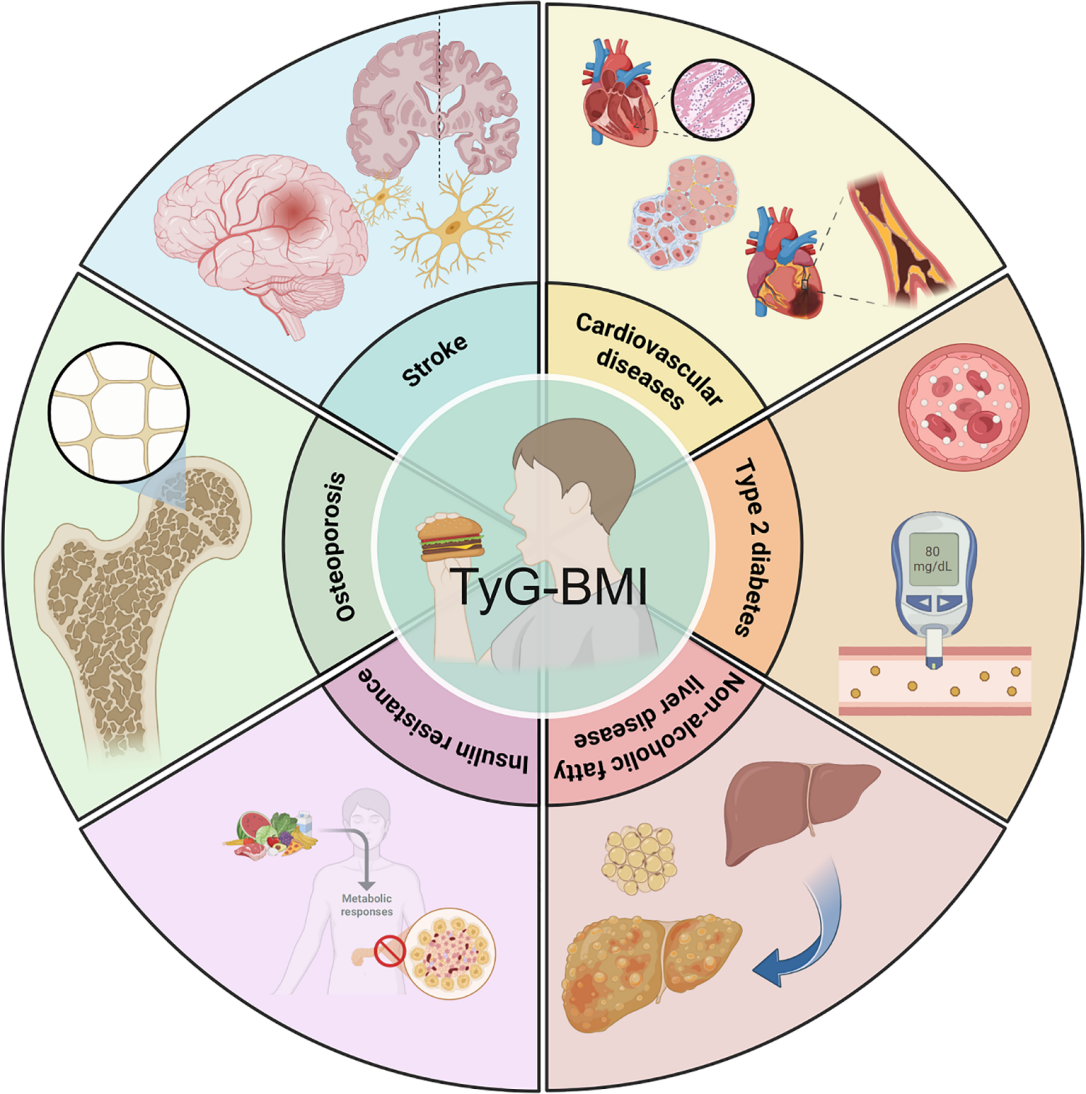
Clinical relevance of TyG-BMI. TyG-BMI is closely associated with type 2 diabetes mellitus, non-alcoholic fatty liver disease, insulin resistance, osteoporosis, stroke, cardiovascular disease, and other metabolic diseases.

TyG-BMI has emerged as an important metric for understanding metabolic and cardiovascular health (**Figure 1**). Insulin resistance (IR) is a key factor underlying many metabolic disorders and TyG-BMI is closely associated with IR (8). In this study involving 511 Taiwanese individuals, various parameters were analyzed to determine their efficiency as independent risk factors for IR. TyG-BMI, calculated as ln [plasma triglyceride (mg/dL) × fasting blood glucose (mg/dL)/2] × BMI, is strongly associated with homeostasis model assessment of insulin resistance (HOMA-IR), with 16.6% of the variability in HOMA-IR explained by TyG-BMI (8). These findings indicate that TyG-BMI can serve as a simple and effective surrogate marker for early identification of IR.

The relationship between TyG-BMI and other metabolic factors is complex. For instance, in a cross-sectional study of 184 overweight/obese individuals without diabetes, TyG-BMI, along with other related parameters, TyG-waist circumference (TyG-WC), was found to be significantly associated with nonalcoholic fatty liver disease (NAFLD) and liver fibrosis (9). The study computed TyG, TyG-BMI, and TyG-WC and determined liver stiffness measurement (LSM) and controlled attenuation parameter (CAP) using transient elastography. Correlation analyses showed that CAP and LSM were significantly associated with WC, BMI, TyG, TyG-BMI, and TyG-WC, suggesting that TyG-BMI is a part of a network of metabolic markers related to adipose tissue and liver health.

## 3 Epidemiological insights

Epidemiological studies have provided valuable insights into the role of TyG-BMI as a cardiovascular risk factor (10–16). In a large-scale analysis of 35,455 participants aged 35–75 years with high risk of cardiovascular disease (CVD) risk, TyG-BMI was associated with an increased risk of all-cause and cardiovascular death (17). The study calculated TyG-BMI as *ln* [fasting triglyceride (mg/dL) × fasting blood glucose (mg/dL)/2] and followed the participants for a median of 3.4 years. Multivariate Cox proportional hazard models showed that participants with a TyG-BMI ≥ 9.83 had a higher risk of all - cause death (Hazard ratio [HR] 1.86, 95% Confidence intervals [CI] 1.37 - 2.51, P < 0.001) and cardiovascular death (HR 2.41, 95%CI 1.47-3.96, P = 0.001) compared to those with a TyG - BMI < 9.83.

Furthermore, in a study of 11,016 US adults from the NHANES 2011–2020 dataset, higher TyG-BMI values were significantly associated with an increased prevalence of CVD (16). Weighted generalized linear models demonstrated a positive association, with individuals in the highest tertile of TyG-BMI having a 38% higher CVD prevalence compared to those in the lowest quartile (OR = 1.380; 95% CI = 1.080, 1.763). These findings suggest that TyG-BMI can be an important epidemiological marker for identifying individuals at high risk of CVD.

## 4 TyG-BMI, insulin resistance and hyperinsulinemia

IR is a complex pathophysiological state in which cells exhibit a reduced response to insulin, leading to elevated blood glucose levels (18). The relationship between TyG–BMI and IR is multifaceted. Obesity, often reflected in a high BMI, is closely associated with IR. Additionally, the distribution and function of adipose tissue play a role; visceral adiposity, which can be related to TyG–BMI, is associated with IR. Moreover, the visceral adiposity index (VAI) and lipid accumulation product (LAP)—indices related to TyG–BMI—are effective surrogate markers for IR and are predictors of MS and DM (19).

In a study of 511 Taiwanese individuals, TyG–BMI was strongly associated with HOMA-IR, with 16.6% of the variability in HOMA-IR explained by TyG–BMI. Receiver operating characteristic (ROC) curve analysis indicated that TyG–BMI had the largest AUC (0.801), suggesting its effectiveness as a surrogate marker for the early identification of IR (20). In a Korean study, the adjusted odds ratio for predicting IR was 12.82 (95% CI: 10.89–15.10) in TyG–BMI quartile 4 compared with quartile 1. The AUC–ROC for TyG–BMI was 0.748, suggesting its superiority over other parameters for IR prediction in the Korean adult population (21).

IR is significantly associated with cardiovascular events and is considered an independent risk factor for CVD (22–24). Multiple studies have shown that IR is closely associated with various cardiovascular risk factors, including hypertriglyceridemia, low high-density lipoprotein cholesterol levels, hypertension, obesity, and elevated plasminogen activator inhibitor-1 levels (25). The combined action of these factors accelerates the development of atherosclerosis and increases the risk of cardiovascular events (26).

IR and hyperinsulinemia play distinct roles in cardiovascular diseases, and their combined effects increase the risk of cardiovascular diseases. IR itself can trigger a series of cardiovascular problems. IR impairs the function of vascular endothelial cells, reduces the production of nitric oxide (NO), increases oxidative stress, leads to decreased vasodilatation ability, and promotes atherosclerosis (27). Furthermore, IR is associated with a chronic low-grade inflammatory state, with elevated levels of inflammatory factors such as interleukin-6 (IL-6) and tumor necrosis factor alpha (TNFα), further exacerbating atherosclerosis (28). Moreover, IR often accompanies dyslipidemia, such as elevated triglycerides and decreased high-density lipoprotein cholesterol (HDL-C), which are risk factors for cardiovascular diseases (29). Long-term hyperinsulinemia can also have adverse effects on the cardiovascular system. Hyperinsulinemia increases renal sodium reabsorption, activates the sympathetic nervous system, and leads to elevated blood pressure (30). Insulin promotes the proliferation of vascular smooth muscle cells, and long-term hyperinsulinemia accelerates the progression of atherosclerosis (31). Hyperinsulinemia may affect the coagulation system, increasing the risk of thrombosis (32). Therefore, IR and hyperinsulinemia each play their own roles in cardiovascular diseases while also influencing each other. Early intervention for IR and hyperinsulinemia is crucial for the prevention and control of cardiovascular diseases.

## 5 Current clinical application

Currently, various diagnostic techniques are used for cardiovascular diseases and TyG-BMI can potentially complement these methods. For example, coronary artery disease is often diagnosed using invasive angiography and computed tomographic angiography. However, these methods have limitations, such as exposure to invasive complications, ionizing radiation, and iodinated contrast agents (33).

Noninvasive methods, such as cardiac magnetic resonance imaging (CMR) angiography, are being explored as alternatives. In addition, TyG-BMI measurements can provide valuable information about the underlying metabolic state of the patient. In a study of patients with ischemia and nonobstructive coronary arteries (INOCA), TyG-BMI was found to be an independent predictor of the slow coronary flow phenomenon (SCFP) (34). This study enrolled 1,625 patients with INOCA and divided them into the SCFP and control groups based on thrombolysis in myocardial infarction (TIMI) frame counts. TyG-BMI was significantly higher in the SCFP group and showed a better predictive value than BMI or triglyceride glucose alone, suggesting that it could be used as an additional diagnostic marker in such patients.

Therapeutic strategies targeting TyG-BMIs are emerging as an important aspect of cardiovascular disease management. Because TyG-BMI is closely associated with insulin resistance, interventions that improve insulin sensitivity may also affect TyG-BMI. Lifestyle modifications such as diet and exercise are fundamental in this regard. A study on the effects of digital health obesity treatment interventions in medically vulnerable primary care patients showed that interventions aimed at weight loss and improving metabolic parameters could potentially influence TyG-BMI-related factors (35).

In terms of pharmacological interventions, drugs that target lipid and glucose metabolism may also affect TyG-BMI. For example, a study on the association between TyG-BMI and the incidence of cardiovascular disease in middle-aged and older Chinese adults suggested that effective management of metabolic risk factors could potentially modify TyG-BMI and reduce CVD risk (36). However, further research is needed to determine the specific drugs and treatment regimens that can most effectively target TyG-BMI and improve cardiovascular outcomes.

Several cardiovascular risk assessment tools are available in clinical practice, and understanding their performance with TyG-BMI is crucial (37). However, these tools may not fully capture the risk associated with insulin resistance, which is represented by TyG-BMI. In a study comparing different risk assessment models in patients with and without rheumatoid arthritis, it was found that the addition of certain biomarkers could improve the predictive accuracy of the models (38). Similarly, incorporating TyG-BMI into existing cardiovascular risk assessment tools may enhance their ability to identify individuals at high risk for cardiovascular diseases. For example, in a study of patients with type H hypertension, TyG-BMI was significantly associated with the severity of coronary artery disease and could potentially be used to refine risk assessments in this patient population (39).

Traditional cardiovascular risk assessment tools typically focus on a single factor, whereas TyG–BMI considered both metabolic and anthropometric factors, thereby improving the predictive accuracy (40). This comprehensive assessment is superior to using BMI or the TyG index alone, as CVD risk is complex and influenced by multiple factors (41). TyG–BMI can help identify early CVD risk factors that traditional risk assessment methods may overlook. For example, adding the TyG index to traditional risk models can improve the model’s ability to distinguish CVD cases (42).

Although TyG–BMI has value in assessing metabolic risk, some limitations remain (43, 44). In summary, while TyG–BMI can serve as an indicator of metabolic risk, it cannot be the sole basis for guiding treatment and preventive drug use. In clinical practice, the overall risk status of patients should be comprehensively considered, and personalized intervention strategies should be developed based on other biomarkers and clinical judgment.

## 6 Controversies and challenges

Despite growing evidence of an association between TyG-BMI and various cardiovascular and metabolic conditions, its clinical utility remains debated. Some studies have questioned the specificity of TyG-BMI as a biomarker (8). Additionally, the cut-off values for TyG-BMI in predicting different diseases may vary across populations studied, which adds to the complexity of its clinical application. Further research is needed to standardize these cutoff values and determine the optimal use of TyG-BMI in different clinical settings.

The integration of TyG-BMI into the clinical practice presents several challenges. One of the main challenges is a lack of awareness among healthcare providers. Many clinicians are not familiar with the concept of TyG-BMI and its potential clinical implications (9).

In addition, there is a need for more standardized measurements and reporting of TyG BMI. Although there is a primary calculation method, TyG-BMI may also have other variations that may be relevant to specific study populations or experimental designs. Different studies may adjust the calculation formula of TyG-BMI according to the needs of the research subjects or experimental design (40). Moreover, the interpretation of TyG-BMI values in the context of other clinical parameters must be better defined. For example, in a study investigating the use of TyG-BMI in predicting the slow coronary flow phenomenon in patients with INOCA, while TyG-BMI was found to be a significant predictor, more research is needed to determine how it should be integrated with other diagnostic and prognostic factors in clinical decision-making (34).

## 7 Future directions

Emerging therapies targeting TyG-BMI are likely to focus on improving insulin sensitivity and modulating lipid and glucose metabolism. One potential approach is the development of drugs that specifically target pathways related to TyG-BMI components. For instance, drugs that reduce triglyceride levels while improving glucose metabolism may have beneficial effects on TyG-BMI. In a study of the association between TyG-BMI and new-onset diabetes, a nonlinear relationship was found, suggesting that interventions aimed at different TyG-BMI levels may be required (45).

TyG-BMI has the potential to play a significant role in personalized medicine for CVH. Personalized treatment plans can be developed by integrating TyG-BMI with other genetic, genomic, and clinical data. For example, in a study on patient similarity-based predictive modeling for cardiovascular diseases, the use of multimodal data, including genetic and clinical information, was explored (46).

Adding TyG-BMI to this data pool could enhance the accuracy of predicting an individual’s risk and treatment response. Additionally, the development of targeted therapies based on TyG-BMI and associated metabolic profiles could be a future direction. This could involve the use of precision medicine approaches, such as pharmacogenetics, to optimize the treatment of cardiovascular diseases in patients with abnormal TyG-BMI.

## References

[1] Zheng Y Ley SH Hu FB. Global aetiology and epidemiology of type 2 diabetes mellitus and its complications. Nat Rev Endocrinol. (2018) 14:88–98. doi: 10.1038/nrendo.2017.151, PMID: 29219149

[2] International Hypoglycaemia Study Group. Hypoglycaemia, cardiovascular disease, and mortality in diabetes: epidemiology, pathogenesis, and management. Lancet Diabetes Endocrinol. (2019) 7:385–96. doi: 10.1016/S2213-8587(18)30315-2, PMID: 30926258

[3] Ortiz-Martínez M González-González M Martagón AJ Hlavinka V Willson RC Rito-Palomares M. Recent developments in biomarkers for diagnosis and screening of type 2 diabetes mellitus. Curr Diabetes Rep. (2022) 22:95–115. doi: 10.1007/s11892-022-01453-4, PMID: 35267140 PMC8907395

[4] Navarro-González D Sánchez-Íñigo L Fernández-Montero A Pastrana-Delgado J Martinez JA. TyG index change is more determinant for forecasting type 2 diabetes onset than weight gain. Med (Baltimore). (2016) 95:e3646. doi: 10.1097/MD.0000000000003646, PMID: 27175686 PMC4902528

[5] Lee DY Lee ES Kim JH Park SE Park CY Oh KW. Predictive value of triglyceride glucose index for the risk of incident diabetes: A 4-year retrospective longitudinal study. PloS One. (2016) 11:e0163465. doi: 10.1371/journal.pone.0163465, PMID: 27682598 PMC5040250

[6] Raimi TH Dele-Ojo BF Dada SA Fadare JO Ajayi DD Ajayi EA. Triglyceride-glucose index and related parameters predicted metabolic syndrome in Nigerians. Metab Syndr Relat Disord. (2021) 19:76–82. doi: 10.1089/met.2020.0092, PMID: 33170086 PMC7929914

[7] Huang W Wang H Shen Z Wang X Yu X. Association between TyG index and risk of carotid atherosclerosis in NAFLD patients: a retrospective cohort study. Front Endocrinol (Lausanne). (2024) 15:1448359. doi: 10.3389/fendo.2024.1448359, PMID: 39229376 PMC11368734

[8] Er LK Wu S Chou HH Hsu LA Teng MS Sun YC. Triglyceride glucose-body mass index is a simple and clinically useful surrogate marker for insulin resistance in nondiabetic individuals. PloS One. (2016) 11:e0149731. doi: 10.1371/journal.pone.0149731, PMID: 26930652 PMC4773118

[9] Khamseh ME Malek M Abbasi R Taheri H Lahouti M Alaei-Shahmiri F. Triglyceride glucose index and related parameters (triglyceride glucose-body mass index and triglyceride glucose-waist circumference) identify nonalcoholic fatty liver and liver fibrosis in individuals with overweight/obesity. Metab Syndr Relat Disord. (2021) 19:167–73. doi: 10.1089/met.2020.0109, PMID: 33259744

[10] Zhang S Liu W Xu B Wang S Du Z Cheng W. Association of triglyceride glucose index and triglyceride glucose-body mass index with sudden cardiac arrest in the general population. Cardiovasc Diabetol. (2024) 23:173. doi: 10.1186/s12933-024-02275-2, PMID: 38762473 PMC11102616

[11] Lyu L Wang X Xu J Liu Z He Y Zhu W. Association between triglyceride glucose-body mass index and long-term adverse outcomes of heart failure patients with coronary heart disease. Cardiovasc Diabetol. (2024) 23:162. doi: 10.1186/s12933-024-02213-2, PMID: 38724999 PMC11080126

[12] Cheng Y Fang Z Zhang X Wen Y Lu J He S. Association between triglyceride glucose-body mass index and cardiovascular outcomes in patients undergoing percutaneous coronary intervention: a retrospective study. Cardiovasc Diabetol. (2023) 22:75. doi: 10.1186/s12933-023-01794-8, PMID: 36997935 PMC10064664

[13] Li W Shen C Kong W Zhou X Fan H Zhang Y. Association between the triglyceride glucose-body mass index and future cardiovascular disease risk in a population with Cardiovascular-Kidney-Metabolic syndrome stage 0–3: a nationwide prospective cohort study. Cardiovasc Diabetol. (2024) 23:292. doi: 10.1186/s12933-024-02352-6, PMID: 39113004 PMC11308445

[14] Li F Wang Y Shi B Sun S Wang S Pang S. Association between the cumulative average triglyceride glucose-body mass index and cardiovascular disease incidence among the middle-aged and older population: a prospective nationwide cohort study in China. Cardiovasc Diabetol. (2024) 23:16. doi: 10.1186/s12933-023-02114-w, PMID: 38184577 PMC10771655

[15] Liu Q Cui H Ma Y Han X Cao Z Wu Y. Triglyceride-glucose index associated with the risk of cardiovascular disease: the Kailuan study. Endocrine. (2022) 75:392–9. doi: 10.1007/s12020-021-02862-3, PMID: 34542800

[16] Wang R Cheng X Tao W. Association between triglyceride glucose body mass index and cardiovascular disease in adults: evidence from NHANES 2011–2020. Front Endocrinol (Lausanne). (2024) 15:1362667. doi: 10.3389/fendo.2024.1362667, PMID: 39081788 PMC11286411

[17] Cai XL Xiang YF Chen XF Lin XQ Lin BT Zhou GY. Prognostic value of triglyceride glucose index in population at high cardiovascular disease risk. Cardiovasc Diabetol. (2023) 22:198. doi: 10.1186/s12933-023-01924-2, PMID: 37537553 PMC10398968

[18] Papaetis GS Sacharidou A Michaelides IC Mikellidis KC Karvounaris SA. Insulin resistance, hyperinsulinemia and atherosclerosis: insights into pathophysiological aspects and future therapeutic prospects. Curr Cardiol Rev. (2025) 21:e1573403X314035. doi: 10.2174/011573403X314035241006185109, PMID: 39415589 PMC12060932

[19] Teng MS Wu S Er LK Hsu LA Chou HH Ko YL. LIPC variants as genetic determinants of adiposity status, visceral adiposity indicators, and triglyceride-glucose (TyG) index-related parameters mediated by serum triglyceride levels. Diabetol Metab Syndr. (2018) 10:79. doi: 10.1186/s13098-018-0383-9, PMID: 30410583 PMC6218991

[20] Zhang S Du T Li M Jia J Lu H Lin X. Triglyceride glucose-body mass index is effective in identifying nonalcoholic fatty liver disease in nonobese subjects. Med (Baltimore). (2017) 96:e7041. doi: 10.1097/MD.0000000000007041, PMID: 28562560 PMC5459725

[21] Lim J Kim J Koo SH Kwon GC. Comparison of triglyceride glucose index, and related parameters to predict insulin resistance in Korean adults: An analysis of the 2007–2010 Korean National Health and Nutrition Examination Survey. PloS One. (2019) 14:e0212963. doi: 10.1371/journal.pone.0212963, PMID: 30845237 PMC6405083

[22] Bigazzi R Bianchi S Buoncristiani E Campese VM. Increased cardiovascular events in hypertensive patients with insulin resistance: a 13-year follow-up. Nutr Metab Cardiovasc Dis. (2008) 18:314–9. doi: 10.1016/j.numecd.2006.11.001, PMID: 17368007

[23] Tenenbaum A Adler Y Boyko V Tenenbaum H Fisman EZ Tanne D. Insulin resistance is associated with increased risk of major cardiovascular events in patients with preexisting coronary artery disease. Am Heart J. (2007) 153:559–65. doi: 10.1016/j.ahj.2007.01.008, PMID: 17383294

[24] Ormazabal V Nair S Elfeky O Aguayo C Salomon C Zuñiga FA. Association between insulin resistance and the development of cardiovascular disease. Cardiovasc Diabetol. (2018) 17:122. doi: 10.1186/s12933-018-0762-4, PMID: 30170598 PMC6119242

[25] Reddy KJ Singh M Bangit JR Batsell RR. The role of insulin resistance in the pathogenesis of atherosclerotic cardiovascular disease: an updated review. J Cardiovasc Med (Hagerstown). (2010) 11:633–47. doi: 10.2459/JCM.0b013e328333645a, PMID: 20164784

[26] Yun JS Ko SH. Current trends in epidemiology of cardiovascular disease and cardiovascular risk management in type 2 diabetes. Metabolism. (2021) 123:154838. doi: 10.1016/j.metabol.2021.154838, PMID: 34333002

[27] van Sloten TT Henry RM Dekker JM Nijpels G Unger T Schram MT. Endothelial dysfunction plays a key role in increasing cardiovascular risk in type 2 diabetes: the Hoorn study. Hypertension. (2014) 64:1299–305. doi: 10.1161/HYPERTENSIONAHA.114.04221, PMID: 25225211

[28] Mancusi C Izzo R di Gioia G Losi MA Barbato E Morisco C. Insulin resistance the hinge between hypertension and type 2 diabetes. High Blood Press Cardiovasc Prev. (2020) 27:515–26. doi: 10.1007/s40292-020-00408-8, PMID: 32964344 PMC7661395

[29] Poznyak A Grechko AV Poggio P Myasoedova VA Alfieri V Orekhov AN. The diabetes mellitus-atherosclerosis connection: the role of lipid and glucose metabolism and chronic inflammation. Int J Mol Sci. (2020) 21:1835. doi: 10.3390/ijms21051835, PMID: 32155866 PMC7084712

[30] Weidmann P Böhlen L de Courten M. Insulin resistance and hyperinsulinemia in hypertension. J Hypertens Suppl. (1995) 13:S65–72. doi: 10.1097/00004872-199508001-00010, PMID: 8576790

[31] Del Turco S Gaggini M Daniele G Basta G Folli F Sicari R. Insulin resistance and endothelial dysfunction: a mutual relationship in cardiometabolic risk. Curr Pharm Des. (2013) 19:2420–31. doi: 10.2174/1381612811319130010, PMID: 23173591

[32] Boden G Rao AK. Effects of hyperglycemia and hyperinsulinemia on the tissue factor pathway of blood coagulation. Curr Diabetes Rep. (2007) 7:223–7. doi: 10.1007/s11892-007-0035-1, PMID: 17547839

[33] Hajhosseiny R Bustin A Munoz C Rashid I Cruz G Manning WJ. Coronary magnetic resonance angiography: technical innovations leading us to the promised land? JACC Cardiovasc Imaging. (2020) 13:2653–72. doi: 10.1016/j.jcmg.2020.01.006, PMID: 32199836

[34] Li ZP Chen J Xin Q Pei XY Wu HL Tan ZX. Triglyceride glucose-body mass index as a novel predictor of slow coronary flow phenomenon in patients with ischemia and nonobstructive coronary arteries (INOCA). BMC Cardiovasc Disord. (2024) 24:60. doi: 10.1186/s12872-024-03722-4, PMID: 38243161 PMC10797862

[35] Foley P Steinberg D Levine E Askew S Batch BC Puleo EM. Track: A randomized controlled trial of a digital health obesity treatment intervention for medically vulnerable primary care patients. Contemp Clin Trials. (2016) 48:12–20. doi: 10.1016/j.cct.2016.03.006, PMID: 26995281 PMC4885789

[36] Bai W Chen H Wan H Ye X Ling Y Xu J. Association between the triglyceride glucose-body roundness index and the incidence of cardiovascular disease among Chinese middle and old-aged adults: a nationwide prospective cohort study. Acta Diabetol. (2025). doi: 10.1007/s00592-025-02499-y, PMID: 40167638

[37] Quispe R Bazo-Alvarez JC Burroughs Peña MS Poterico JA Gilman RH Checkley W. Distribution of short-term and lifetime predicted risks of cardiovascular diseases in Peruvian adults. J Am Heart Assoc. (2015) 4:e002112. doi: 10.1161/JAHA.115.002112, PMID: 26254303 PMC4599468

[38] Alemao E Cawston H Bourhis F Al M Rutten-van Molken M Liao KP. Comparison of cardiovascular risk algorithms in patients with vs without rheumatoid arthritis and the role of C-reactive protein in predicting cardiovascular outcomes in rheumatoid arthritis. Rheumatol (Oxf Engl). (2017) 56:777–86. doi: 10.1093/rheumatology/kew440, PMID: 28087832 PMC8344293

[39] Wang L Li Z Qiu R Luo L Yan X. Triglyceride glucose index-body mass index as a predictor of coronary artery disease severity in patients with H-type hypertension across different glucose metabolic states. Diabetol Metab Syndr. (2025) 17:15. doi: 10.1186/s13098-024-01568-6, PMID: 39815366 PMC11734521

[40] Song K Xu Y Wu S Zhang X Wang Y Pan S. Research status of triglyceride glucose-body mass index (TyG-BMI index). Front Cardiovasc Med. (2025) 12:1597112. doi: 10.3389/fcvm.2025.1597112, PMID: 40756600 PMC12315701

[41] Cheng Y Wu S Chen S Wu Y. Association of body mass index combined with triglyceride-glucose index in cardiovascular disease risk: a prospective cohort study. Sci Rep. (2025) 15:17687. doi: 10.1038/s41598-025-02342-y, PMID: 40399481 PMC12095778

[42] Chen Q Xiong S Zhang Z Yu X Chen Y Ye T. Triglyceride-glucose index is associated with recurrent revascularization in patients with type 2 diabetes mellitus after percutaneous coronary intervention. Cardiovasc Diabetol. (2023) 22:284. doi: 10.1186/s12933-023-02011-2, PMID: 37865753 PMC10590524

[43] Wang H He S Wang J Qian X Zhang B Yang Z. Assessing and predicting type 2 diabetes risk with triglyceride glucose-body mass index in the Chinese nondiabetic population-Data from long-term follow-up of Da Qing IGT and Diabetes Study. J Diabetes. (2024) 16:e70001. doi: 10.1111/1753-0407.70001, PMID: 39364793 PMC11450669

[44] Lima do Vale MR Buckner L Mitrofan CG Tramontt CR Kargbo SK Khalid A. A synthesis of pathways linking diet, metabolic risk and cardiovascular disease: a framework to guide further research and approaches to evidence-based practice. Nutr Res Rev. (2023) 36:232–58. doi: 10.1017/S0954422421000378, PMID: 34839838

[45] Qiao Q Liang K Wang C Wang L Yan F Chen L. J-shaped association of the triglyceride glucose-body mass index with new-onset diabetes. Sci Rep. (2024) 14:13882. doi: 10.1038/s41598-024-64784-0, PMID: 38880800 PMC11180648

[46] Sharafoddini A Dubin JA Lee J. Patient similarity in prediction models based on health data: A scoping review. JMIR Med Inform. (2017) 5:e7. doi: 10.2196/medinform.6730, PMID: 28258046 PMC5357318

